# Crystal structure, DFT calculations and Hirshfeld surface analysis of 3-(4-methyl­phen­yl)-6-nitro-1*H*-indazole

**DOI:** 10.1107/S205698901801647X

**Published:** 2018-11-22

**Authors:** Ali Ben-Yahia, Youness El Bakri, Chin-Hung Lai, El Mokhtar Essassi, Joel T. Mague

**Affiliations:** a Laboratoire de Chimie Organique Hétérocyclique, Centre de Recherche des Sciences des Médicaments, URAC 21, Pôle de Compétence Pharmacochimie, Av Ibn Battouta, BP 1014, Faculté des Sciences, Université Mohammed V, Rabat, Morocco; b Department of Chemistry, Peoples’ Friendship University of Russia (RUDN University), 6 Miklukho-Maklaya St, Moscow 117198, Russian Federation; c Department of Medical Applied Chemistry, Chung Shan Medical University, Taichung 40241, Taiwan; d Department of Chemistry, Tulane University, New Orleans, LA 70118, USA

**Keywords:** crystal structure, indazole, hydrogen bonds, π-stacking

## Abstract

The asymmetric unit of the title compound, C_14_H_11_N_3_O_3_, consists of two independent mol­ecules having very similar conformations in which the indazole moieties are planar. The independent mol­ecules are distinguished by small differences in the rotational orientations of the nitro groups. In the crystal, N—H⋯O and C—H⋯O hydrogen bonds form zigzag chains along the *b*-axis direction. Additional C—H⋯O hydrogen bonds link the chains into layers parallel to (10). These are connected by slipped π-stacking and C—H⋯π(ring) inter­actions.

## Abstract 2

The asymmetric unit of the title compound consist of two independent mol­ecules. In the crystal, N–H⋯O and C—H⋯O hydrogen bonds form zigzag chains along the *b*-axis direction. Additional C—H⋯O hydrogen bonds link the chains into layers parallel to (10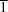). These are connected by slipped π-stacking and C—H⋯π(ring) inter­actions.

## Chemical context   

Indazoles are an important class of heterocyclic compounds having a wide range of biological and pharmaceutical applications. There is enormous potential in the synthesis of novel heterocyclic systems to be used as building blocks for the next generation of pharmaceuticals as anti-bacterial, anti-depressant and anti-inflammatory agents. Fused aromatic 1*H* and 2*H*-indazoles are well recognized for their anti-hypertensive and anti-cancer properties while other indazole derivatives are a versatile class of compounds that have found use in biology, catalysis and medicinal chemistry (Schmidt *et al.*, 2008 ▸). Although rare in nature (Liu *et al.*, 2004 ▸; Ali *et al.*, 2008 ▸), indazoles exhibit a variety of biological activities such as HIV protease inhibition (Patel *et al.*, 1999 ▸), anti­arrhythmic and analgesic activities (Mosti *et al.*, 2000 ▸) and anti­tumor activity and anti­hypertensive properties (Bouissane *et al.*, 2006 ▸; Abbassi *et al.*, 2012 ▸). As a continuation of our studies of indazole derivatives (Mohamed Abdelahi *et al.*, 2017*a* ▸,*b* ▸,*c* ▸), we report the synthesis and structure of the title compound, (I).
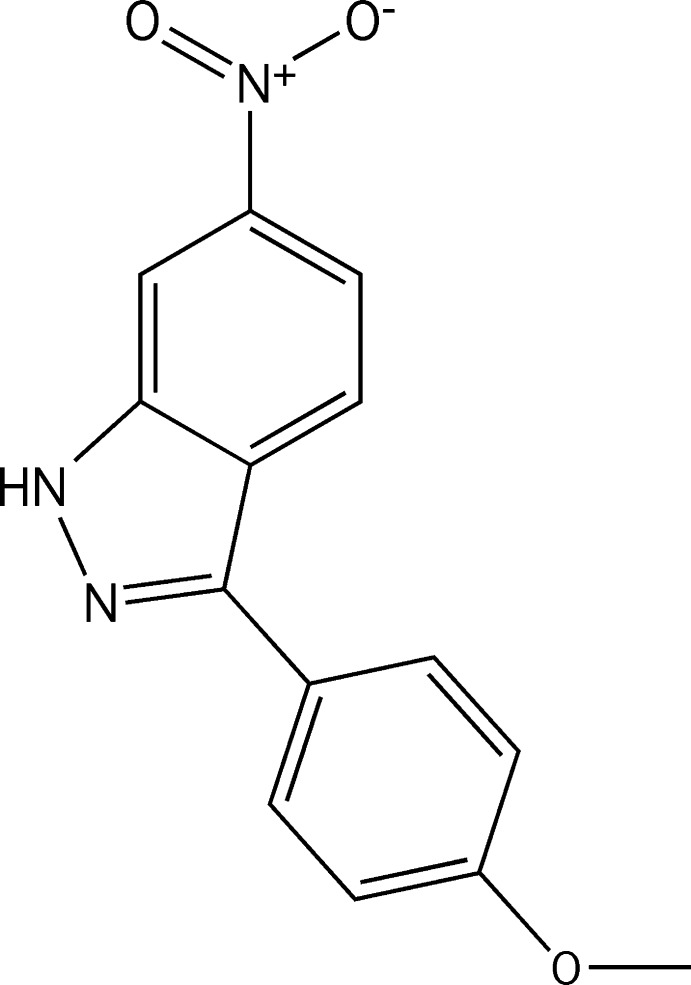


## Structural commentary   

**Figure 1:**
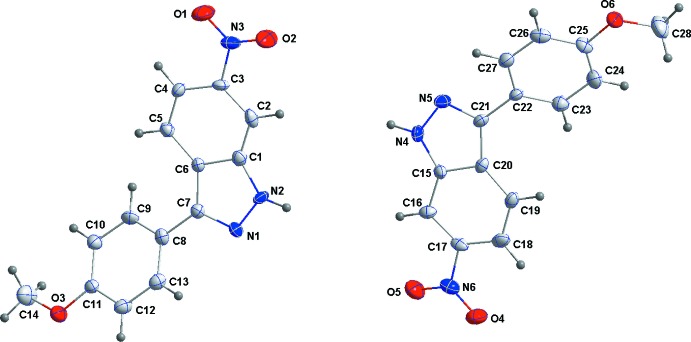
The asymmetric unit of (I) with the labelling scheme and 50% probability ellipsoids.

The asymmetric unit of (I) consists of two independent mol­ecules differing only slightly in conformation (Fig. 1 ▸, Table 1). The largest difference is in the twist of the nitro group as indicated by the torsion angles O2—N3—C3—C2 and O5—N6—C17—C16 which are −1.1 (9) and 4.0 (9)°, respectively. In the mol­ecule containing N1, the indazole portion is planar to within 0.045 (6) Å (r.m.s. deviation = 0.007 Å) and the C8–C13 ring is inclined to this plane by 30.8 (3)°. In the mol­ecule containing N4, the indazole portion is planar to within 0.036 (5) Å (r.m.s. deviation = 0.007 Å) and the C22–C27 ring is inclined to this plane by 31.6 (3)°.

## Supra­molecular features   

**Figure 2:**
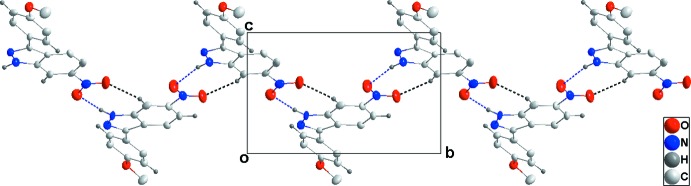
Detail of one zigzag chain in (I) viewed along the *a*-axis direction. N—H⋯O and C—H⋯O hydrogen bonds are shown, respectively, by blue and black dashed lines.

**Figure 3:**
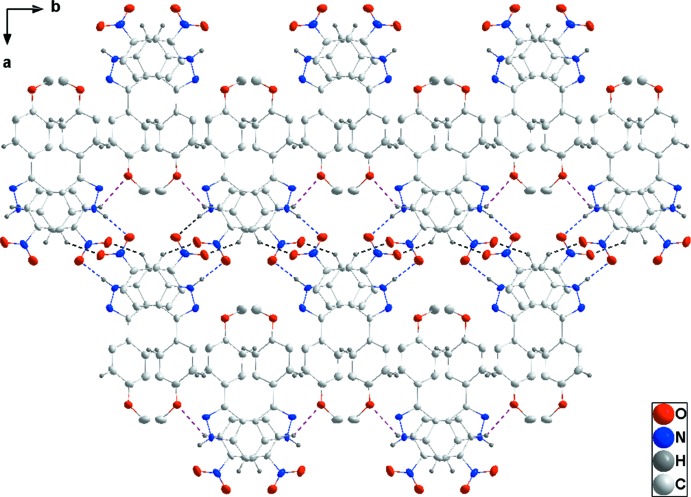
Plan view of the layer structure of (I) seen along the *c*-axis direction. Portions of one chain extend horizontally with the intra­chain hydrogen bonds depicted as in Fig. 2 ▸. The C—H⋯O hydrogen bonds connecting the chains into layers are depicted by purple dashed lines.

**Figure 4:**
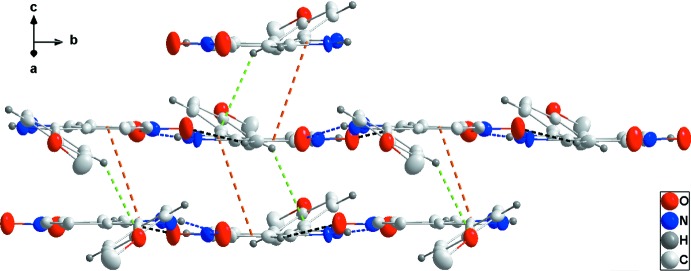
Elevation view of the layer structure of (I) projected on (401). π-stacking and C—H⋯π(ring) inter­actions are shown, respectively, by orange and green dashed lines. Hydrogen bonds are depicted as in Fig. 2 ▸.

In the crystal of (I), alternating N2—H2*A*⋯O5 and N4—H4*A*⋯O2 hydrogen bonds coupled with C16—H16⋯O1 hydrogen bonds form zigzag chains extending along the *b*-axis direction (Table 1 ▸ and Fig. 2 ▸). These chains are connected into layers parallel to (10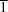) by C4—H4⋯O1 hydrogen bonds (Table 1 ▸ and Fig. 3 ▸). The layers bound to one another by a combination of slipped π-stacking inter­actions between the C1–C6 and N1/N2/C1/C6/C7 rings [centroid–centroid distance = 3.699 (4) Å, dihedral angle = 2.4 (4)°] and between the N4/N5/C21/C20/C15 and C15–C20 rings [centroid–centroid distance= 3.636 (4) Å, dihedral angle = 2.6 (4)°]. These are reinforced by the C—H⋯π(ring) inter­actions (C10—H10⋯*Cg*3, C13—H13⋯*Cg*7, C23—H23⋯*Cg*7 and C26—H26⋯*Cg*3; Table 1 ▸ and Fig. 4 ▸).

## Database survey   

A search of the Cambridge Structural Database (Version 5.39; Groom *et al.*, 2016 ▸) found 70 structures of indazoles not containing a substituent on the secondary nitro­gen atom and not ligands in metal complexes. Of these, only seven are nitro derivatives. These are 3,7-di­nitro­indazole (Cabildo *et al.*, 2011 ▸), two determinations of 7-nitro­indazole (Ooms *et al.*, 2000 ▸; Sopková-de Oliveira Santos *et al.*, 2000 ▸), 7-nitro-1*H*-indazol-3-ol (Claramunt *et al.*, 2009 ▸), 3-(4-methyl­phen­yl)-6-nitro-1*H*-indazole (Liu *et al.*, 2014 ▸) and 5-nitro-3-thio­morpholino-1*H*-indazole and 5-nitro-3-(4-methyl­piper­az­ino)-1*H*-indazole (Gzella & Wrzeciono, 2001 ▸). The structures of the nitro derivatives are fairly similar to that in the present work in that the indazole moieties are essentially planar with the nitro groups twisted out the plane by 3–6°. In the 4-methyl­phenyl derivative, the phenyl ring is inclined to the plane of the indazole moiety by 12.94 (8)°.

## DFT calculations and Hirshfeld surface analysis   

### DFT calculations   

The structure of the title compound in the gas phase was optimized by means of density functional theory. The DFT calculation was performed by the hybrid B3LYP method, which is based on the idea of Becke and considers a mixture of the exact (HF) and DFT exchange utilizing the B3 functional together with the LYP correlation functional (Becke, 1993 ▸; Lee *et al.*, 1988 ▸; Miehlich *et al.*, 1989 ▸). The B3LYP calculation was performed in conjunction with a triple-*x* basis set which was designed for the DFT optimization [designated as TZVP (DFT orbital); Godbout *et al.*, 1992 ▸]. After obtaining the converged geometry, the harmonic vibrational frequencies were calculated at the same theoretical level to confirm that the number of the imaginary frequency is zero for the stationary point. Both the geometry optimization and harmonic vibrational frequency analysis of the title compound were carried out with the *Gaussian16* program (Frisch *et al.*, 2016 ▸).

### Hirshfeld surface calculations   

Both the definition of a mol­ecule in a condensed phase and the recognition of distinct entities in mol­ecular liquids and crystals are fundamental concepts in chemistry. Based on Hirshfeld’s partitioning scheme, a method to divide the electron distribution in a crystalline phase into mol­ecular fragments was proposed (Spackman & Byrom, 1997 ▸; McKinnon *et al.*, 2004 ▸; Spackman & Jayatilaka, 2009 ▸). This partitioned the crystal into regions where the electron distribution of a sum of spherical atoms for the mol­ecule dominates over the corresponding sum of the crystal. Because it derived from Hirshfeld’s stockholder partitioning, the mol­ecular surface is named the Hirshfeld surface. In this study, the Hirshfeld surface analysis of the title compound was performed using the *CrystalExplorer* program (Turner *et al.*, 2017 ▸).

### theoretical comparison of the title compound   

**Figure 5:**
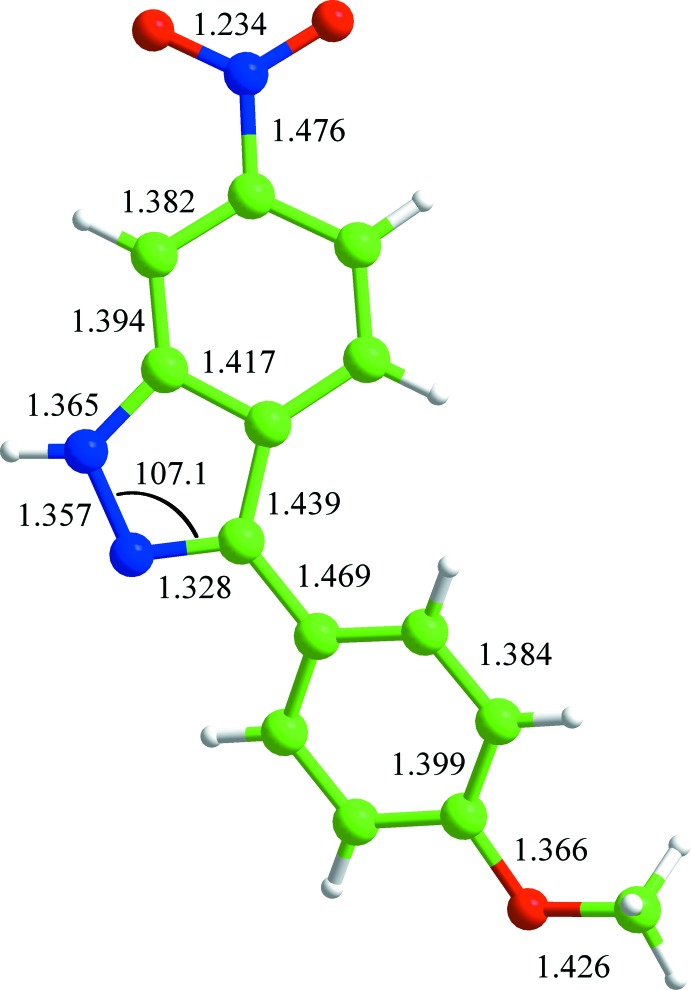
The B3LYP-optimized geometries (Å,*°*) of (I).

The results of the B3LYP geometry optimization of (I) are depicted in Fig. 5 ▸ and a comparative study of the gas-phase structure and the solid-phase one for (I) was performed, with the results summarized in Table 2 ▸ together with a previous geometrical study on 1*H*-indazole itself (Hathaway *et al.*, 1998 ▸). The discrepancy between our B3LYP result and the previous MP2(fc) calculations may be due to the substitutent effects of both the NO_2_ and meth­oxy­phenyl groups (Hathaway *et al.*, 1998 ▸).

### Hirshfeld analysis of the title compound   

**Figure 6:**
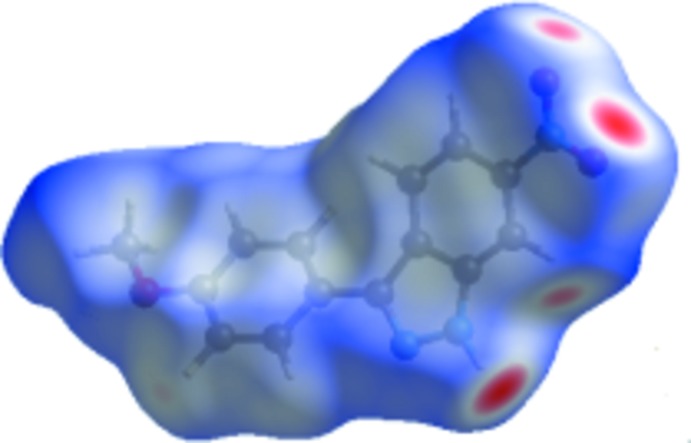
The *d*_norm_ Hirshfeld surface of (I) (red: negative, white: zero, blue: positive; scale: −0.4664–1.4050 a.u.).

The standard resolution mol­ecular Hirshfeld surface (*d*_norm_) of the title compound is shown in Fig. 6 ▸ and is transparent so the mol­ecular moiety can be visualized in a similar orientation for all of the structures around which they were calculated. The 3D *d*_norm_ surface can be used to identify very close inter­molecular inter­actions with *d*_norm_ being negative (positive) when inter­molecular contacts are shorter (longer) than the sum of the van der Waals radii. The *d*_norm_ value is mapped onto the Hirshfeld surface by red, white or blue colours. The red regions represent closer contacts with a negative *d*_norm_ while the blue regions represent longer contacts with a positive *d*_norm_ and the white regions represent contacts equal to the van der Waals separation with *d*_norm_ equal to zero. As depicted in Fig. 6 ▸, the major inter­actions in the title compound are the inter­molecular H⋯O and H⋯N hydrogen bonds.

**Figure 7:**
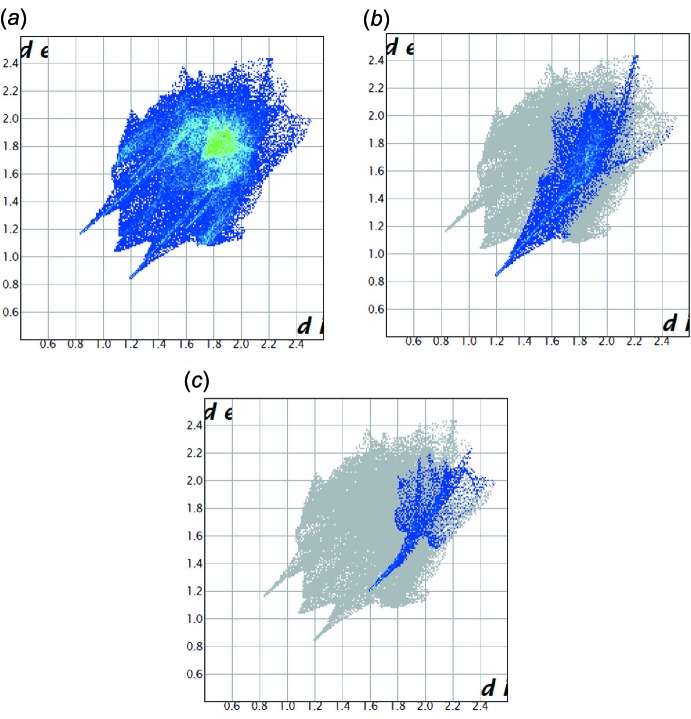
Two-dimensional fingerprint plots of (I): (*a*) full, (*b*) resolved into H⋯O contacts; (*c*) resolved into H⋯N contacts.

The 2D fingerprint plots highlight particular atom-pair contacts and enable the separation of contributions from different inter­action types that overlap in the full fingerprint. Using the standard 0.6–2.6 Å view with the *d*_e_ and *d*_i_ distance scales displayed on the graph axes, the 2D fingerprint plot for the title compound is shown in Fig. 7 ▸(*a*). Including the recip­rocal contacts, the contribution of the O⋯H contacts (15.7%) for the title compound is larger than that of the N⋯H contacts (4.6%) [Fig. 7 ▸(*b*) and 7(*c*)].

## Synthesis and crystallization   

**6-Nitro-3-(4-meth­oxy­phen­yl)-1** ***H*** **-indazole (I):**

To a solution of 6-nitro­indazole (0.1 g) dissolved in 1.5 mL of a mixture of 1,4-dioxane/EtOH (3/1, *v*/*v*) in a microwave tube with a stir bar were added *p*-meth­oxy­phenyl­boronic acid (1.5 equiv.), a solution of caesium carbonate (1.3 equiv.) dissolved in 0.5 mL of H_2_O and Pd(PPh_3_)_4_ (0.1 equiv.) under argon. The reaction vessel was sealed with a silicone septum and was subjected to microwave irradiation at 413 K with stirring. The reaction mixture was then allowed to cool to room temperature, diluted with ethyl acetate (15 mL) and water (10 mL) and extracted (3 times). The combined organic layer was dried over MgSO_4_ and concentrated under reduced pressure. The crude material was purified by column chromatography on silica gel (EtOAc/Ether) to give the desired final product. Yield: 74%. Orange solid, m.p. 503–505 K. ^1^H NMR (400 MHz, DMSO-*d_6_*) δ 13.74 (*s*, 1H), 8.46 (*d*, *J* = 1.5 Hz, 1H), 8.24 (*d*, *J* = 9.0 Hz, 1H), 7.96 (*dd*, *J* = 1.5, 9.0 Hz, 1H), 7.92 (*d*, *J* = 8.6 Hz, 2H), 7.10 (*d*, *J* = 8.6 Hz, 2H), 3.82 (3H, *s*). ^13^C NMR (100 MHz, DMSO-*d_6_*) δ 159.8, 146.1, 144.2, 140.7, 128.7, 125.3, 123.3, 122.4, 115.5, 114.9, 107.8, 55.6. HRMS (ESI) *m*/*z* calculated for C_14_H_11_N_3_O_3_ [*M* + H]^+^: 270.0834, found 270.0780.

## Refinement   

Crystal data, data collection and structure refinement details are summarized in Table 3 ▸. H atoms attached to carbon were placed in calculated positions (C—H = 0.95–0.98 Å) while those attached to nitro­gen were placed in locations derived from a difference map and their parameters adjusted to give N—H = 0.91 Å. All were included as riding contributions with *U*_iso_(H) = 1.2–1.5*U*_eq_(C,N).

## Supplementary Material

Crystal structure: contains datablock(s) global, I. DOI: 10.1107/S205698901801647X/ff2156sup1.cif

Structure factors: contains datablock(s) I. DOI: 10.1107/S205698901801647X/ff2156Isup2.hkl

Supporting information file. DOI: 10.1107/S205698901801647X/ff2156Isup3.cdx

Supporting information file. DOI: 10.1107/S205698901801647X/ff2156Isup4.cml

CCDC reference: 1879920

Additional supporting information:  crystallographic information; 3D view; checkCIF report

## Figures and Tables

**Table 1:** Hydrogen-bond geometry (Å, °)

*D*—H⋯*A*	*D*—H	H⋯*A*	*D*⋯*A*	*D*—H⋯*A*
N2—H2*A*⋯O5^i^	0.90	2.11	3.005 (9)	173
C2—H2⋯O4^i^	0.95	2.41	3.201 (10)	140
C4—H4⋯O6^ii^	0.95	2.60	3.329 (9)	134
N4—H4*A*⋯O2^iii^	0.91	2.15	3.043 (9)	168
C16—H16⋯O1^iii^	0.95	2.39	3.171 (10)	139
C18—H18⋯O3^iv^	0.95	2.61	3.340 (10)	134

Symmetry codes: (i) 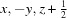; (ii) 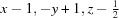; (iii) 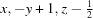; (iv) 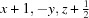.

**Table 2:** The B3LYP-optimized and the X-ray structural parameters (Å, °) for (I)

	B3LYP	X-ray	1*H*-indazole^*a*^
N1—N2	1.357	1.358 (8)	1.349
N1—C7	1.328	1.323 (9)	1.337
N2—C1	1.365	1.363 (10)	1.367
C1—C2	1.394	1.378 (11)	1.406
C1—C6	1.417	1.404 (10)	1.422
C2—C3	1.328	1.368 (9)	1.389
C3—C4	1.408	1.410 (11)	1.419
C4—C5	1.380	1.370 (10)	1.388
C5—C6	1.405	1.420 (10)	1.412
C6—C7	1.439	1.438 (10)	1.424
C7—N1—N2	107.1	106.8 (7)	105.5

Note: (*a*) MP2(fc)/6–311G** calculated values (Hathaway *et al.*, 1998 ▸).

**Table 3:** Experimental details

Crystal data	
Chemical formula	C_14_H_11_N_3_O_3_
*M*_r_	269.26
Crystal system, space group	Monoclinic, *P* *c*
Temperature (K)	180
*a*, *b*, *c* (Å)	14.1447 (14), 11.8380 (12), 7.4252 (8)
β (°)	96.681 (7)
*V* (Å^3^)	1234.9 (2)
*Z*	4
Radiation type	Mo *K*α
μ (mm^−1^)	0.11
Crystal size (mm)	0.18 × 0.02 × 0.02

Data collection	
Diffractometer	Bruker *SMART* *APEX*
Absorption correction	Multi-scan (*SADABS*; Bruker, 2016 ▸)
*T*_min_, *T*_max_	0.70, 0.75
No. of measured, independent and observed [*I* > 2σ(*I*)] reflections	25051, 6667, 2803
*R*_int_	0.111
(sin θ/λ)_max_ (Å^−1^)	0.715

Refinement	
*R*[*F* ^2^ > 2σ(*F* ^2^)], *wR*(*F* ^2^), *S*	0.064, 0.158, 0.94
No. of reflections	6667
No. of parameters	363
No. of restraints	2
H-atom treatment	H-atom parameters constrained
Δρ_max_, Δρ_min_ (e Å^−3^)	0.46, −0.32
Absolute structure	Flack *x* determined using 891 quotients [(*I* ^+^)−(*I* ^−^)]/[(*I* ^+^)+(*I* ^−^)] (Parsons *et al.*, 2013 ▸)
Absolute structure parameter	0.6 (10)

Computer programs: *APEX3* and *SAINT* (Bruker, 2016 ▸), *SHELXT* (Sheldrick, 2015*a* ▸), *SHELXL2018* (Sheldrick, 2015*b* ▸), *DIAMOND* (Brandenburg & Putz, 2012 ▸) and *SHELXTL* (Sheldrick, 2008 ▸).

## supplementary crystallographic information

### Crystal data

**Table:**

C_14_H_11_N_3_O_3_	*F*(000) = 560
*M**_r_* = 269.26	*D*_x_ = 1.448 Mg m^−^^3^
Monoclinic, *P**c*	Mo *K*α radiation, λ = 0.71073 Å
*a* = 14.1447 (14) Å	Cell parameters from 1994 reflections
*b* = 11.8380 (12) Å	θ = 3.4–24.8°
*c* = 7.4252 (8) Å	µ = 0.11 mm^−^^1^
β = 96.681 (7)°	*T* = 180 K
*V* = 1234.9 (2) Å^3^	Needle, orange
*Z* = 4	0.18 × 0.02 × 0.02 mm

### Data collection

**Table:**

Bruker SMART APEX diffractometer	6667 independent reflections
Radiation source: fine-focus sealed tube	2803 reflections with *I* > 2σ(*I*)
Graphite monochromator	*R*_int_ = 0.111
Detector resolution: 8.333 pixels mm^-1^	θ_max_ = 30.5°, θ_min_ = 1.7°
ω–φ scans	*h* = −20→19
Absorption correction: multi-scan (SADABS; Bruker, 2016)	*k* = −16→16
*T*_min_ = 0.70, *T*_max_ = 0.75	*l* = −10→10
25051 measured reflections	

### Refinement

**Table:**

Refinement on *F*^2^	Secondary atom site location: difference Fourier map
Least-squares matrix: full	Hydrogen site location: mixed
*R*[*F*^2^ > 2σ(*F*^2^)] = 0.064	H-atom parameters constrained
*wR*(*F*^2^) = 0.158	*w* = 1/[σ^2^(*F*_o_^2^) + (0.0622*P*)^2^] where *P* = (*F*_o_^2^ + 2*F*_c_^2^)/3
*S* = 0.94	(Δ/σ)_max_ < 0.001
6667 reflections	Δρ_max_ = 0.46 e Å^−^^3^
363 parameters	Δρ_min_ = −0.32 e Å^−^^3^
2 restraints	Absolute structure: Flack *x* determined using 891 quotients [(*I*^+^)-(*I*^-^)]/[(*I*^+^)+(*I*^-^)] (Parsons *et al.*, 2013)
Primary atom site location: structure-invariant direct methods	Absolute structure parameter: 0.6 (10)

### Special details

**Table:**

Geometry. All esds (except the esd in the dihedral angle between two l.s. planes) are estimated using the full covariance matrix. The cell esds are taken into account individually in the estimation of esds in distances, angles and torsion angles; correlations between esds in cell parameters are only used when they are defined by crystal symmetry. An approximate (isotropic) treatment of cell esds is used for estimating esds involving l.s. planes.
Refinement. H-atoms attached to carbon were placed in calculated positions (C—H = 0.95 - 0.98 Å) while those attached to nitrogen were placed in locations derived from a difference map and their parameters adjusted to give N—H = 0.91 Å. All were included as riding contributions with isotropic displacement parameters 1.2 - 1.5 times those of the attached atoms.

### Fractional atomic coordinates and isotropic or equivalent isotropic displacement parameters (Å^2^)

**Table:**

	*x*	*y*	*z*	*U*_iso_*/*U*_eq_	
O1	0.4650 (5)	0.7694 (5)	0.4740 (8)	0.0490 (17)	
O2	0.5455 (5)	0.6197 (5)	0.5579 (8)	0.0492 (19)	
O3	−0.2022 (4)	0.3751 (4)	−0.0943 (6)	0.0305 (14)	
N1	0.2293 (4)	0.2766 (6)	0.2357 (7)	0.0253 (17)	
H2A	0.3518	0.2443	0.3613	0.030*	
N2	0.3179 (4)	0.3039 (6)	0.3141 (7)	0.0248 (15)	
N3	0.4744 (5)	0.6668 (7)	0.4797 (9)	0.0311 (16)	
C1	0.3319 (5)	0.4178 (7)	0.3224 (9)	0.0237 (18)	
C2	0.4084 (5)	0.4805 (7)	0.3985 (9)	0.0259 (19)	
H2	0.4654	0.4459	0.4523	0.031*	
C3	0.3978 (6)	0.5954 (7)	0.3925 (9)	0.0231 (17)	
C4	0.3146 (6)	0.6497 (6)	0.3121 (9)	0.0234 (18)	
H4	0.3106	0.7298	0.3115	0.028*	
C5	0.2396 (5)	0.5862 (6)	0.2349 (9)	0.0221 (18)	
H5	0.1834	0.6216	0.1790	0.026*	
C6	0.2473 (5)	0.4667 (7)	0.2400 (8)	0.0180 (17)	
C7	0.1848 (6)	0.3728 (6)	0.1920 (9)	0.019 (2)	
C8	0.0853 (6)	0.3749 (7)	0.1091 (9)	0.021 (2)	
C9	0.0491 (4)	0.4629 (5)	−0.0003 (8)	0.0244 (14)	
H9	0.0904	0.5223	−0.0269	0.029*	
C10	−0.0468 (4)	0.4668 (5)	−0.0732 (9)	0.0260 (14)	
H10	−0.0702	0.5277	−0.1490	0.031*	
C11	−0.1067 (6)	0.3808 (6)	−0.0330 (9)	0.0205 (19)	
C12	−0.0721 (4)	0.2916 (5)	0.0784 (8)	0.0281 (15)	
H12	−0.1135	0.2324	0.1054	0.034*	
C13	0.0229 (4)	0.2896 (5)	0.1498 (8)	0.0248 (14)	
H13	0.0459	0.2293	0.2274	0.030*	
C14	−0.2404 (5)	0.4616 (6)	−0.2163 (10)	0.0406 (18)	
H14A	−0.2092	0.4586	−0.3273	0.061*	
H14B	−0.2291	0.5356	−0.1586	0.061*	
H14C	−0.3090	0.4499	−0.2464	0.061*	
O4	0.5210 (5)	−0.2692 (5)	0.0879 (8)	0.0461 (16)	
O5	0.4437 (4)	−0.1201 (5)	−0.0121 (8)	0.0434 (18)	
O6	1.1756 (4)	0.1266 (4)	0.7138 (6)	0.0322 (14)	
N4	0.6648 (4)	0.1951 (6)	0.2571 (7)	0.0262 (15)	
H4A	0.6277	0.2544	0.2145	0.031*	
N5	0.7533 (4)	0.2231 (6)	0.3387 (7)	0.0252 (17)	
N6	0.5133 (5)	−0.1670 (8)	0.0758 (8)	0.0323 (17)	
C15	0.6528 (5)	0.0827 (6)	0.2389 (9)	0.0195 (16)	
C16	0.5754 (5)	0.0192 (6)	0.1567 (9)	0.0234 (18)	
H16	0.5186	0.0528	0.0999	0.028*	
C17	0.5892 (6)	−0.0958 (7)	0.1658 (9)	0.0236 (17)	
C18	0.6700 (6)	−0.1506 (7)	0.2516 (10)	0.0273 (19)	
H18	0.6730	−0.2307	0.2574	0.033*	
C19	0.7451 (6)	−0.0852 (6)	0.3273 (9)	0.0239 (18)	
H19	0.8013	−0.1198	0.3848	0.029*	
C20	0.7376 (5)	0.0320 (7)	0.3184 (8)	0.0185 (17)	
C21	0.7974 (6)	0.1252 (6)	0.3794 (9)	0.021 (2)	
C22	0.8960 (6)	0.1244 (6)	0.4662 (9)	0.019 (2)	
C23	0.9577 (5)	0.0380 (5)	0.4298 (9)	0.0273 (14)	
H23	0.9348	−0.0213	0.3501	0.033*	
C24	1.0518 (5)	0.0361 (5)	0.5068 (8)	0.0284 (15)	
H24	1.0929	−0.0232	0.4787	0.034*	
C25	1.0852 (6)	0.1210 (7)	0.6245 (9)	0.024 (2)	
C26	1.0248 (4)	0.2077 (5)	0.6667 (8)	0.0267 (15)	
H26	1.0479	0.2652	0.7496	0.032*	
C27	0.9311 (4)	0.2099 (5)	0.5878 (8)	0.0261 (14)	
H27	0.8903	0.2695	0.6159	0.031*	
C28	1.2406 (5)	0.0424 (7)	0.6675 (11)	0.0450 (19)	
H28A	1.2483	0.0484	0.5384	0.067*	
H28B	1.2159	−0.0326	0.6926	0.067*	
H28C	1.3024	0.0535	0.7398	0.067*	

### Atomic displacement parameters (Å^2^)

**Table:**

	*U*^11^	*U*^22^	*U*^33^	*U*^12^	*U*^13^	*U*^23^
O1	0.049 (4)	0.028 (5)	0.067 (4)	−0.012 (4)	−0.006 (3)	−0.007 (3)
O2	0.035 (4)	0.038 (4)	0.067 (4)	−0.006 (3)	−0.023 (3)	−0.001 (3)
O3	0.024 (3)	0.030 (3)	0.036 (3)	−0.001 (2)	−0.005 (2)	0.004 (2)
N1	0.016 (3)	0.026 (5)	0.032 (3)	0.000 (3)	−0.002 (3)	0.001 (3)
N2	0.027 (4)	0.012 (3)	0.033 (3)	−0.001 (3)	−0.004 (3)	0.004 (3)
N3	0.032 (4)	0.026 (4)	0.036 (4)	−0.012 (4)	0.006 (3)	−0.003 (4)
C1	0.023 (4)	0.024 (5)	0.024 (4)	0.001 (4)	0.003 (3)	0.002 (3)
C2	0.018 (4)	0.034 (6)	0.025 (4)	0.007 (4)	0.001 (3)	0.007 (3)
C3	0.022 (4)	0.019 (4)	0.028 (4)	−0.009 (4)	0.000 (3)	0.000 (3)
C4	0.025 (4)	0.015 (4)	0.030 (4)	0.002 (4)	−0.001 (3)	0.001 (3)
C5	0.019 (4)	0.024 (5)	0.023 (4)	0.000 (4)	0.000 (3)	−0.001 (3)
C6	0.016 (4)	0.018 (4)	0.020 (3)	0.001 (4)	0.002 (3)	0.000 (3)
C7	0.019 (5)	0.021 (6)	0.016 (4)	0.000 (3)	0.001 (4)	0.002 (3)
C8	0.019 (5)	0.026 (6)	0.017 (4)	0.003 (3)	0.002 (4)	0.000 (3)
C9	0.026 (3)	0.025 (3)	0.024 (3)	−0.005 (3)	0.006 (3)	0.003 (3)
C10	0.024 (4)	0.024 (3)	0.029 (3)	−0.001 (3)	−0.003 (3)	0.000 (3)
C11	0.020 (5)	0.019 (5)	0.023 (4)	−0.001 (3)	0.003 (3)	−0.003 (3)
C12	0.031 (4)	0.023 (4)	0.029 (3)	−0.004 (3)	−0.004 (3)	0.004 (3)
C13	0.029 (4)	0.023 (3)	0.021 (3)	0.000 (3)	−0.002 (3)	0.003 (3)
C14	0.035 (4)	0.042 (4)	0.041 (4)	0.000 (3)	−0.012 (3)	0.014 (4)
O4	0.042 (4)	0.028 (5)	0.067 (4)	−0.010 (4)	−0.001 (3)	−0.005 (3)
O5	0.029 (4)	0.045 (5)	0.053 (4)	−0.004 (3)	−0.009 (3)	0.001 (3)
O6	0.023 (3)	0.030 (3)	0.041 (3)	−0.001 (2)	−0.008 (2)	−0.004 (2)
N4	0.020 (4)	0.024 (4)	0.034 (3)	0.007 (3)	−0.003 (3)	0.001 (3)
N5	0.026 (4)	0.021 (4)	0.027 (3)	−0.005 (3)	−0.001 (3)	−0.001 (3)
N6	0.027 (4)	0.037 (5)	0.032 (4)	−0.010 (4)	0.003 (3)	−0.004 (3)
C15	0.016 (4)	0.020 (5)	0.023 (3)	0.001 (4)	0.003 (3)	−0.005 (3)
C16	0.026 (4)	0.020 (5)	0.024 (4)	−0.005 (4)	0.001 (3)	−0.004 (3)
C17	0.022 (4)	0.028 (5)	0.021 (3)	−0.009 (4)	0.004 (3)	−0.010 (3)
C18	0.033 (5)	0.022 (4)	0.029 (4)	−0.006 (4)	0.013 (4)	−0.002 (4)
C19	0.027 (5)	0.019 (4)	0.026 (4)	0.004 (4)	0.004 (3)	0.005 (3)
C20	0.024 (4)	0.017 (4)	0.015 (3)	0.003 (4)	0.002 (3)	0.000 (3)
C21	0.026 (6)	0.014 (6)	0.023 (4)	−0.001 (4)	0.002 (4)	0.002 (3)
C22	0.022 (5)	0.013 (5)	0.022 (4)	−0.002 (3)	0.002 (4)	−0.002 (3)
C23	0.027 (4)	0.026 (4)	0.028 (3)	−0.002 (3)	0.001 (3)	−0.003 (3)
C24	0.027 (4)	0.032 (4)	0.027 (3)	0.003 (3)	0.007 (3)	−0.002 (3)
C25	0.021 (5)	0.032 (6)	0.018 (4)	−0.003 (4)	−0.005 (4)	0.001 (3)
C26	0.032 (4)	0.030 (4)	0.018 (3)	−0.010 (3)	0.002 (3)	−0.002 (3)
C27	0.030 (4)	0.020 (3)	0.029 (3)	0.000 (3)	0.005 (3)	−0.001 (3)
C28	0.024 (4)	0.054 (5)	0.054 (5)	0.004 (3)	−0.005 (3)	−0.010 (4)

### Geometric parameters (Å, º)

**Table:**

O1—N3	1.222 (8)	O4—N6	1.217 (9)
O2—N3	1.233 (9)	O5—N6	1.248 (8)
O3—C11	1.377 (9)	O6—C25	1.372 (9)
O3—C14	1.431 (7)	O6—C28	1.425 (8)
N1—C7	1.323 (9)	N4—C15	1.346 (10)
N1—N2	1.358 (8)	N4—N5	1.367 (8)
N2—C1	1.363 (10)	N4—H4A	0.9100
N2—H2A	0.9007	N5—C21	1.334 (9)
N3—C3	1.465 (10)	N6—C17	1.463 (10)
C1—C2	1.378 (11)	C15—C16	1.407 (10)
C1—C6	1.404 (10)	C15—C20	1.408 (10)
C2—C3	1.368 (9)	C16—C17	1.375 (10)
C2—H2	0.9500	C16—H16	0.9500
C3—C4	1.410 (11)	C17—C18	1.401 (12)
C4—C5	1.370 (10)	C18—C19	1.380 (11)
C4—H4	0.9500	C18—H18	0.9500
C5—C6	1.420 (10)	C19—C20	1.393 (10)
C5—H5	0.9500	C19—H19	0.9500
C6—C7	1.438 (10)	C20—C21	1.431 (10)
C7—C8	1.470 (11)	C21—C22	1.467 (12)
C8—C9	1.381 (9)	C22—C23	1.392 (9)
C8—C13	1.398 (9)	C22—C27	1.407 (9)
C9—C10	1.402 (8)	C23—C24	1.385 (8)
C9—H9	0.9500	C23—H23	0.9500
C10—C11	1.378 (9)	C24—C25	1.379 (9)
C10—H10	0.9500	C24—H24	0.9500
C11—C12	1.395 (9)	C25—C26	1.394 (9)
C12—C13	1.385 (8)	C26—C27	1.385 (7)
C12—H12	0.9500	C26—H26	0.9500
C13—H13	0.9500	C27—H27	0.9500
C14—H14A	0.9800	C28—H28A	0.9800
C14—H14B	0.9800	C28—H28B	0.9800
C14—H14C	0.9800	C28—H28C	0.9800
			
C11—O3—C14	117.2 (5)	C25—O6—C28	116.2 (6)
C7—N1—N2	106.8 (7)	C15—N4—N5	112.5 (6)
N1—N2—C1	112.1 (6)	C15—N4—H4A	131.8
N1—N2—H2A	113.8	N5—N4—H4A	115.3
C1—N2—H2A	133.6	C21—N5—N4	105.7 (7)
O1—N3—O2	123.0 (9)	O4—N6—O5	122.7 (9)
O1—N3—C3	119.1 (8)	O4—N6—C17	119.0 (8)
O2—N3—C3	117.9 (8)	O5—N6—C17	118.3 (8)
N2—C1—C2	130.9 (7)	N4—C15—C16	130.9 (7)
N2—C1—C6	106.0 (7)	N4—C15—C20	106.7 (6)
C2—C1—C6	123.0 (7)	C16—C15—C20	122.4 (7)
C3—C2—C1	116.3 (8)	C17—C16—C15	114.1 (8)
C3—C2—H2	121.8	C17—C16—H16	122.9
C1—C2—H2	121.8	C15—C16—H16	122.9
C2—C3—C4	123.4 (8)	C16—C17—C18	125.7 (8)
C2—C3—N3	119.0 (8)	C16—C17—N6	117.1 (8)
C4—C3—N3	117.5 (7)	C18—C17—N6	117.2 (8)
C5—C4—C3	119.6 (7)	C19—C18—C17	118.3 (7)
C5—C4—H4	120.2	C19—C18—H18	120.8
C3—C4—H4	120.2	C17—C18—H18	120.8
C4—C5—C6	118.8 (8)	C18—C19—C20	119.2 (8)
C4—C5—H5	120.6	C18—C19—H19	120.4
C6—C5—H5	120.6	C20—C19—H19	120.4
C1—C6—C5	118.8 (8)	C19—C20—C15	120.1 (8)
C1—C6—C7	104.9 (7)	C19—C20—C21	135.6 (7)
C5—C6—C7	136.1 (7)	C15—C20—C21	104.3 (7)
N1—C7—C6	110.1 (7)	N5—C21—C20	110.8 (7)
N1—C7—C8	121.5 (7)	N5—C21—C22	120.0 (7)
C6—C7—C8	128.4 (7)	C20—C21—C22	129.1 (7)
C9—C8—C13	118.1 (7)	C23—C22—C27	118.1 (7)
C9—C8—C7	122.0 (7)	C23—C22—C21	120.3 (6)
C13—C8—C7	119.7 (7)	C27—C22—C21	121.6 (6)
C8—C9—C10	121.8 (6)	C24—C23—C22	121.7 (6)
C8—C9—H9	119.1	C24—C23—H23	119.2
C10—C9—H9	119.1	C22—C23—H23	119.2
C11—C10—C9	118.9 (6)	C25—C24—C23	119.5 (6)
C11—C10—H10	120.6	C25—C24—H24	120.3
C9—C10—H10	120.6	C23—C24—H24	120.3
O3—C11—C10	124.7 (6)	O6—C25—C24	125.0 (7)
O3—C11—C12	115.0 (6)	O6—C25—C26	114.7 (7)
C10—C11—C12	120.4 (7)	C24—C25—C26	120.3 (7)
C13—C12—C11	119.9 (6)	C27—C26—C25	120.0 (6)
C13—C12—H12	120.1	C27—C26—H26	120.0
C11—C12—H12	120.1	C25—C26—H26	120.0
C12—C13—C8	120.9 (6)	C26—C27—C22	120.4 (6)
C12—C13—H13	119.6	C26—C27—H27	119.8
C8—C13—H13	119.6	C22—C27—H27	119.8
O3—C14—H14A	109.5	O6—C28—H28A	109.5
O3—C14—H14B	109.5	O6—C28—H28B	109.5
H14A—C14—H14B	109.5	H28A—C28—H28B	109.5
O3—C14—H14C	109.5	O6—C28—H28C	109.5
H14A—C14—H14C	109.5	H28A—C28—H28C	109.5
H14B—C14—H14C	109.5	H28B—C28—H28C	109.5
			
C7—N1—N2—C1	0.1 (7)	C15—N4—N5—C21	−2.4 (7)
N1—N2—C1—C2	176.2 (7)	N5—N4—C15—C16	−176.9 (6)
N1—N2—C1—C6	−1.4 (7)	N5—N4—C15—C20	1.9 (7)
N2—C1—C2—C3	−176.4 (7)	N4—C15—C16—C17	−179.6 (6)
C6—C1—C2—C3	0.9 (10)	C20—C15—C16—C17	1.8 (9)
C1—C2—C3—C4	−0.7 (10)	C15—C16—C17—C18	1.8 (10)
C1—C2—C3—N3	176.7 (6)	C15—C16—C17—N6	−177.3 (5)
O1—N3—C3—C2	179.9 (7)	O4—N6—C17—C16	−176.7 (7)
O2—N3—C3—C2	−1.1 (9)	O5—N6—C17—C16	4.0 (9)
O1—N3—C3—C4	−2.5 (9)	O4—N6—C17—C18	4.2 (9)
O2—N3—C3—C4	176.5 (6)	O5—N6—C17—C18	−175.1 (6)
C2—C3—C4—C5	−0.1 (10)	C16—C17—C18—C19	−3.2 (11)
N3—C3—C4—C5	−177.6 (6)	N6—C17—C18—C19	175.8 (6)
C3—C4—C5—C6	0.8 (10)	C17—C18—C19—C20	0.9 (10)
N2—C1—C6—C5	177.6 (6)	C18—C19—C20—C15	2.4 (10)
C2—C1—C6—C5	−0.2 (10)	C18—C19—C20—C21	179.5 (7)
N2—C1—C6—C7	2.0 (6)	N4—C15—C20—C19	177.2 (6)
C2—C1—C6—C7	−175.8 (6)	C16—C15—C20—C19	−3.9 (10)
C4—C5—C6—C1	−0.6 (9)	N4—C15—C20—C21	−0.7 (6)
C4—C5—C6—C7	173.2 (6)	C16—C15—C20—C21	178.2 (5)
N2—N1—C7—C6	1.3 (7)	N4—N5—C21—C20	1.8 (7)
N2—N1—C7—C8	−177.9 (6)	N4—N5—C21—C22	178.7 (6)
C1—C6—C7—N1	−2.1 (7)	C19—C20—C21—N5	−178.2 (7)
C5—C6—C7—N1	−176.5 (7)	C15—C20—C21—N5	−0.7 (7)
C1—C6—C7—C8	177.0 (6)	C19—C20—C21—C22	5.3 (12)
C5—C6—C7—C8	2.6 (11)	C15—C20—C21—C22	−177.2 (7)
N1—C7—C8—C9	−153.8 (7)	N5—C21—C22—C23	−147.3 (7)
C6—C7—C8—C9	27.2 (10)	C20—C21—C22—C23	28.9 (10)
N1—C7—C8—C13	30.2 (9)	N5—C21—C22—C27	32.7 (10)
C6—C7—C8—C13	−148.8 (7)	C20—C21—C22—C27	−151.0 (7)
C13—C8—C9—C10	−1.4 (9)	C27—C22—C23—C24	−1.4 (10)
C7—C8—C9—C10	−177.4 (6)	C21—C22—C23—C24	178.7 (6)
C8—C9—C10—C11	0.5 (9)	C22—C23—C24—C25	0.9 (9)
C14—O3—C11—C10	3.6 (9)	C28—O6—C25—C24	5.4 (9)
C14—O3—C11—C12	−177.0 (6)	C28—O6—C25—C26	−177.1 (6)
C9—C10—C11—O3	179.4 (6)	C23—C24—C25—O6	177.7 (6)
C9—C10—C11—C12	0.0 (9)	C23—C24—C25—C26	0.4 (10)
O3—C11—C12—C13	−179.2 (6)	O6—C25—C26—C27	−178.7 (5)
C10—C11—C12—C13	0.2 (9)	C24—C25—C26—C27	−1.1 (9)
C11—C12—C13—C8	−1.1 (9)	C25—C26—C27—C22	0.6 (8)
C9—C8—C13—C12	1.7 (9)	C23—C22—C27—C26	0.6 (9)
C7—C8—C13—C12	177.8 (6)	C21—C22—C27—C26	−179.5 (6)

### Hydrogen-bond geometry (Å, º)

**Table:**

*D*—H···*A*	*D*—H	H···*A*	*D*···*A*	*D*—H···*A*
N2—H2*A*···O5^i^	0.90	2.11	3.005 (9)	173
C2—H2···O4^i^	0.95	2.41	3.201 (10)	140
C4—H4···O6^ii^	0.95	2.60	3.329 (9)	134
N4—H4*A*···O2^iii^	0.91	2.15	3.043 (9)	168
C16—H16···O1^iii^	0.95	2.39	3.171 (10)	139
C18—H18···O3^iv^	0.95	2.61	3.340 (10)	134

Symmetry codes: (i) *x*, −*y*, *z*+1/2; (ii) *x*−1, −*y*+1, *z*−1/2; (iii) *x*, −*y*+1, *z*−1/2; (iv) *x*+1, −*y*, *z*+1/2.
